# Interaction between maternally derived antibodies and heterogeneity in exposure combined to determine time-to-first *Plasmodium falciparum* infection in Kenyan infants

**DOI:** 10.1186/s12936-019-2657-6

**Published:** 2019-01-22

**Authors:** Arnold Reynaldi, Arlene E. Dent, Timothy E. Schlub, Sidney Ogolla, Rosemary Rochford, Miles P. Davenport

**Affiliations:** 1 Kirby Institute for Infection and Immunity, UNSW Australia, Sydney, NSW 2052 Australia; 2 Center for Global Health and Diseases, Case Western Reserve University, Cleveland, OH USA; 3 Faculty of Medicine and Health, Sydney School of Public Health, The University of Sydney, Sydney, NSW 2006 Australia; 4 Centre for Global Health Research, Kenya Medical Research Institute, Kisumu, Kenya; 5 Department of Immunology and Microbiology, University of Colorado, Denver, CO USA

**Keywords:** *Plasmodium falciparum* malaria, Antibody, Immunity, Heterogeneity in exposure, Newborns

## Abstract

**Background:**

Studies of the association between the level of anti-malarial antibody and protection from malaria infection can yield conflicting results if they fail to take into account differences in the malaria transmission rate. This can occur because high malaria exposure may drive high antibody responses, leading to an apparent positive association between immune response and infection rate. The neonatal period provides a unique window to study the protective effects of antibodies, because waning maternally-derived antibodies lead to different levels of protection with time.

**Methods:**

This study uses data from two well-defined infant cohorts in Western Kenya with different burdens of malaria transmission. Survival models were used to assess how the magnitude of maternally derived malaria-specific IgG antibody (to 24 malaria antigens measured using Luminex beads) affected the time-to-first *Plasmodium falciparum* infection (detected by PCR). In addition, mathematical models were used to assess how the frequency of malaria infection varied between the cohorts with different exposure levels.

**Results:**

Despite differences in underlying malaria incidence in the two regions, there was no difference in time-to-first malaria infection between the cohorts. However, there was a significant period of protection observed in children with high initial MSP1 (42 kDa fragment)-specific antibody levels, but this protection was not observed in children with low antibody levels. Children from the high transmission cohort had both longer initial periods of protection from malaria (attributable to higher initial antibody levels), but more rapid time-to-first-infection once malaria specific maternal antibodies declined below protective levels (attributable to higher exposure rates).

**Conclusion:**

This study demonstrates the complex interaction between passive (maternally-derived) immunity and the degree of malaria exposure in infants. Children from regions of high malaria transmission had higher levels of maternally-derived antibodies in early life, which led to a significant protection for several months. However, once this immunity waned, the underlying higher frequency of infection was revealed. A better understanding of the interaction between malaria exposure, immunity, and transmission risk will assist in identifying protective immune responses in *P. falciparum* infection.

**Electronic supplementary material:**

The online version of this article (10.1186/s12936-019-2657-6) contains supplementary material, which is available to authorized users.

## Background

The identification of *Plasmodium falciparum* antigens that are targets of naturally-acquired protective antibodies has been the goal of many studies on malaria immunoepidemiology [1–5]. A common method for identifying protective immune responses is the prospective study of individuals with different initial levels of anti-malarial immune responses, to identify those who are protected from subsequent malaria. However, if individuals vary significantly in their exposure to infection [6, 7], high levels of exposure may induce high levels of antibody responses, and thus sero-reactivity can sometimes be observed paradoxically as a risk factor for malaria [8, 9]. This may explain why identification of protective immune responses may be more difficult than expected [10].

The trans-placental transfer of maternal IgG antibodies to infants plays a critical role in protection of infants from common childhood infections including malaria [11]. Importantly, it is not only the presence but the levels of maternal antibodies in infants that correlate with protection from malaria [12, 13] (reviewed in [14]). This suggests that neonatal antibody levels and malaria exposure present a unique opportunity to investigate the role of naturally acquired (maternal) antibody in protection from *P. falciparum* infection. That is, mothers may vary in their levels of exposure to *P. falciparum* infection and hence their levels of anti-malarial antibody. Similarly, it is expected that maternal malaria exposure is likely a predictor of infant exposure. Thus, children of highly exposed mothers may initially experience both high exposure and high protection (from maternal antibodies). However, as maternal antibody protection wanes with time, early immune protection may be followed by a period of reducing antibody levels and increased susceptibility to infection. This may provide a ‘window’ in which the underlying level of exposure can be assessed in the absence of interference from different levels of maternal acquired immunity.

In this study, the interactions between antibody levels, exposure, and time-to-first infection were investigated. This study was based on the previous longitudinal study of malaria-specific antibody levels and frequency of *P. falciparum* DNA detection (indicative of infection) in two well-defined infant cohorts in Kenya with different intensities of malaria transmission [15, 16]. Given the higher exposure in infants from Kisumu (the high malaria transmission region), the time-to-first-infection was expected to be shorter in this area than in nearby Nandi (the low transmissions area). Surprisingly, the time-to-first-infection with malaria was not significantly different between the two cohorts, despite the expected differences in malaria exposure. Analysis and modelling of antibody levels and exposure reveals that this similar time-to-first infection in the two regions was due to the complex interactions between exposure, passive immunity from maternal antibodies, and waning antibody levels with age.

## Methods

### Study participants

Two cohorts of children were established at the Chulaimbo Sub-District Hospital in Kisumu County and at the Mosoriot Sub-District Hospital in Nandi County, Kenya. These cohorts will be referred to as malaria^hi^ (i.e., from stable/high malaria transmission region) and malaria^lo^ (i.e., from unstable/low malaria transmission region). Both serve patients from a predominantly rural area. Mothers of newborn infants who were attending post-natal clinics were approached for participation in the study. Enrollment from both sites occurred in between April and May 2006 and infants from both sites were followed simultaneously to eliminate any effects of seasonality in malaria exposures. The description of this longitudinal cohort was previously reported [16]. Inclusion criteria were infants of any gender born to HIV-negative mothers. Ethical approval for this study was provided by the Kenya Medical Research Institute Ethical Review Committee and the Institutional Review Board at State University of New York Upstate Medical University (site where study was initiated) and at the University of Colorado, Denver. The first sampling and data collection were performed when the children were approximately 1 month of age (Additional file 1), then sampling was performed every month through the first 12 months, and every 4 months after that through 2 years of age. Infants with greater than a 3-month gap in samples were excluded from the study.

### Measurement of anti-malaria IgG antibodies

The following *P. falciparum* antigens were used in a Luminex multiplex assay (performed in the laboratory of A.E. Dent, Cleveland, OH): liver stage antigen 1 (LSA1); circumsporozoite protein (CSP); cell-traversal protein for ookinetes and sporozoites (PfCelTOS); serine repeat antigen 5 (SERA5); RIPR CT (C terminus) and NT (N terminus); reticulocyte binding protein-like homologue (Rh2 and Rh4.9); merozoite surface protein (MSP2-FC27, MSP6, MSP7, MSP3, MSPDBL1, MSPDBL2); MSP1 42-kDa fragment (MSP1_42_ 3D7, FVO, and FUP)(referred to as MSP1 for simplicity); erythrocyte binding antigen 140 (EBA 140), EBA175, and EBA181; and apical membrane antigen 1 (AMA1 3D7 and FVO). Proteins were conjugated to non-magnetic microsphere bioplex beads (Bio-Rad) according to the manufacturer’s instructions with slight modifications. Briefly, varying concentrations of each protein were coupled to beads to obtain an optimal antibody signal in a linear range against a serially diluted positive control pool of plasma from healthy malaria immune adult residents of Western Kenya. Plasma was incubated with conjugated beads at a 1:1 ratio for final dilutions of 1:100 and 1:1000 to select the appropriate dilution within the linear range of the positive control pool. The secondary antibody was R-Phycoerythrin AffiniPure F(ab’)2 fragment goat anti-human IgG F(ab’)2 (Jackson ImmunoResearch Laboratories). A more detailed description of the use of this assay to test *P. falciparum* antibodies has been described previously [17]. Mean fluorescent intensity (MFI) values obtained from the assay and MFI values greater than the mean MFI + 3 standard deviations of malaria naïve controls (from US adult controls) were considered positive.

### Detection of *Plasmodium falciparum* DNA by PCR

DNA was extracted from up to 200 µl blood using Qiagen DNAeasy kit (Qiagen, Valencia, CA) according to the manufacturers’ protocol. DNA was eluted off the column in an equivalent volume of H_2_0 and stored at − 20 °C. *Plasmodium falciparum* DNA was detected using PCR primer and probes as described [18]. *Plasmodium falciparum* DNA was quantified by including a TopoII plasmid that contained the *P. falciparum* PCR product to generate a standard curve. The sensitivity of this assay was 4 copies of *P. falciparum* DNA/ml.

### Statistical methods

Hazard ratios (HRs) for time-to-first infection between regions and for antibody level was measured with a cox proportional hazards model. Three different Cox models were used throughout this study. First, a standard Cox model was used to investigate if the level of antibody at first detection is associated with time to infection (Additional file 2). Then, a time-dependent Cox model was used to investigate if the level of antibody at any time (which changes with time) is a significant determinant of time to infection. Finally, a model with a ‘protective threshold’ of antibody level was also used. Antibody threshold levels were identified using the *segmented* package in *R* [19]. Then, having found the threshold value (*Thr*), the significance was assessed by using a time-dependent Cox regression model in the form of:

1 $$ \begin{aligned} & \beta_{1} A_{i} \left( t \right) + \beta_{2} Prot + \beta_{3} age \\  &  Prot = \left\{ {\begin{array}{ll}    {0,} & {A_{i} \left( t \right) < Thr}  \\    {A_{i} \left( t \right) - Thr,} & {A_{i} \left( t \right) \ge Thr}  \\   \end{array} } \right.  \\ \end{aligned} $$

where A_i_(t) is the antibody level of child *i* at time *t*. Thus, the hazard ratio for antibody level increases by $e^{{\beta_{1} }}$ for every 1 unit increase in antibody level before the threshold is reached; and increases by $e^{{\beta_{1} + \beta_{2} }}$ after the threshold. Thus, $e^{{\beta_{2} }}$ gives the fold-increase (or decrease) in hazard ratio before and after the threshold and its significance tests the level of evidence in support of a threshold. Thus, this test asks whether the level of antibody affects the probability of getting infected, and also if the probability of infection is different between children with antibody levels below a threshold, and those with antibody above the threshold). P-values for HRs were based on the likelihood ratio tests.

Differences in antibody levels between US adult controls, infants from the malaria^lo^ setting (Nandi), and infants from the malaria^hi^ setting (Kisumu) were analysed with the non-parametric Mann–Whitney *U* test. These analyses were carried out in *R: A language and environment for statistical computing* (version 3.3.3).

### Modelling the kinetics of MSP1 IgG

Levels of inherited maternal antibody levels ($A$) were modelled with a fixed decay rate $\delta$ (exponential decay), until the levels reach a plateau level *B*. This can be written as:

2 $$ M\left( t \right) = Ae^{ - \delta  t} + B $$

To account for within child correlations due to the longitudinal nature of the data, the model was fitted using a non-linear mixed effect model using *R* function *nlme* in library *nlme* (v3.1-122), with fixed effects for A, $\delta$ and B, and random effects for A and B. 95% CI was calculated using *R* function *intervals* in library *nlme*. In this study, the initial antibody levels in children from Nandi were not significantly different from that of unexposed controls, and the level was fixed to the baseline value *B*.

### Modelling the time-to-first *P. falciparum* DNA detection

The time-to-first malaria infection in different populations was modelled using a parametric exponential survival with an added delay term as follow:

3 $$  S\left( t \right) = \left\{ {\begin{array}{ll}    {1,} & {t < T_{{on}} }  \\    {e^{{ - k\left( {t - T_{{on}} } \right)}} ,} & {t \ge T_{{on}} }  \\   \end{array} } \right.  $$

where $k$ is the infection rate, and $T_{on}$ is the time delay to infection risk due to maternally inherited immunity. Then, the differences in *T*_*on*_ and *k* between high and low malaria groups were also tested (using an F-test).

## Results

### Time-to-first *P. falciparum* infection in high versus low malaria regions

**Fig. 1:**
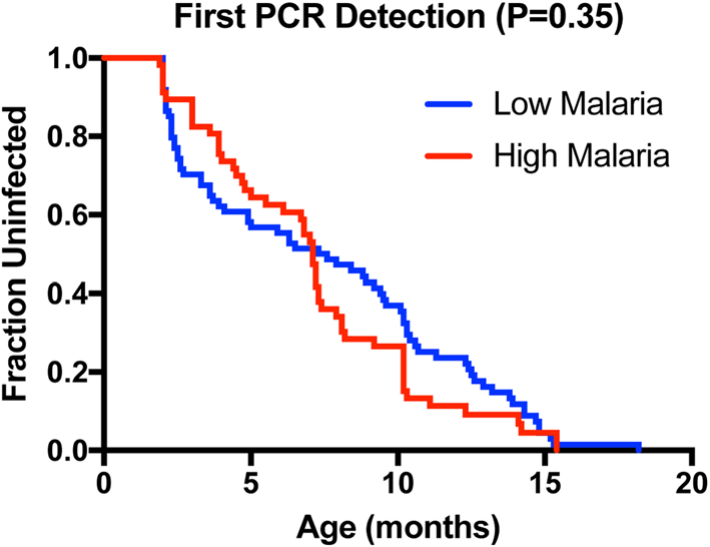
Time-to-first *Plasmodium falciparum* infection. There is no difference in the time-to-infection (defined by the presence of DNA in peripheral blood) between children in Kisumu (high malaria) and Nandi (low malaria). Despite malaria transmission being lower in Nandi, it seems that many children were infected earlier in this region compared to Kisumu (*P* value for proportional hazard assumption P = 0.017, test for the trend in the weighted residuals)

Previous analyses of the same cohort have shown that children in malaria^hi^ cohort experienced more malaria infections than children in malaria^lo^ cohort [15, 16]. In this study, infection was defined as the presence of *P. falciparum* DNA in peripheral blood (as measured by qPCR). Given the higher malaria infection rate in malaria^hi^, it is expected that time-to-first infection with *P. falciparum* should be shorter in this cohort. However, unexpectedly there was no significant difference in the time to detection of *P. falciparum* DNA between the malaria^hi^ and malaria^lo^ groups (P = 0.35, log rank test; Fig. 1; Additional files 3 and 4—for the number of people at risk in high and low malaria region). Despite no overall difference across the study period, close observation of Fig. 1 reveals that a greater proportion of infants in the low malaria region became infected in the first 6 months of life and infants from the high malaria region experienced more infection later (P = 0.017 for interaction between hazard ratio and time, leading the survival curves to cross). To alleviate this, a Cox model was used to split the data into two periods (occurrence of infection in less than 6 months vs more than 6 months). The first 6 months period showed a trend towards protection for children in malaria^hi^ group (compared with the malaria^lo^ group)(HR = 0.59 for children living in malaria^hi^ region relative to malaria^lo^ region, P-value = 0.062, 95% CI = [0.33–1.03]). However, the reverse was the case after 6 months, as children form the malaria^hi^ group showed higher infection rates (HR = 1.79 for children in malaria^hi^ region relative to malaria^lo^ region, p-value = 0.019, 95% CI = [1.1–2.91]). That is, there is evidence for a difference in time-to-first infection before/after 6 months which, when averaged over the entire study period, leads to no observable effect. This first 6 months coincides with a period of maximum protection due to maternal antibodies [20]. Thus, this pattern of infection risk may be caused by differences in initial (maternally derived) antibody levels between the cohorts.

### Detection of *P. falciparum*-specific IgG in infants

To investigate the possibility that the pattern of infection risk was governed by immune protection, antibody binding to 24 malaria antigens were measured using a Luminex multiplex bead-based array [17]. Antibodies were screened using a number of criteria to focus on those most likely to be important in protection. First, the levels of various antibodies at first detection were tested to see if they were different between the two exposed cohorts (living in malaria^hi^ and malaria^lo^ region) and uninfected US adult controls. Then, the levels at first detection of various antibodies were also tested to see if they were also different between the malaria^hi^ and malaria^lo^ group. Ultimately, the levels of various antibodies were also analysed to see if they could be used to predict the time to first malaria infection (detectable by PCR). The results are summarized in Additional files 2 and 5. Interestingly, only one antigen—MSP1—was significantly different between infected cohort and uninfected US adult controls (also different between children in malaria^hi^ vs malaria^lo^ group). This was also correlated with the time to first malaria infection. Thus, the analysis will be focused on the two allelic forms measured in this study—3D7 and FVO. Antibody levels to both antigen alleles were highly correlated, thus only 3D7 results are presented (MSP1-FVO—Additional files 6 and 7).

### Antibody dynamics and protection

**Fig. 2:**
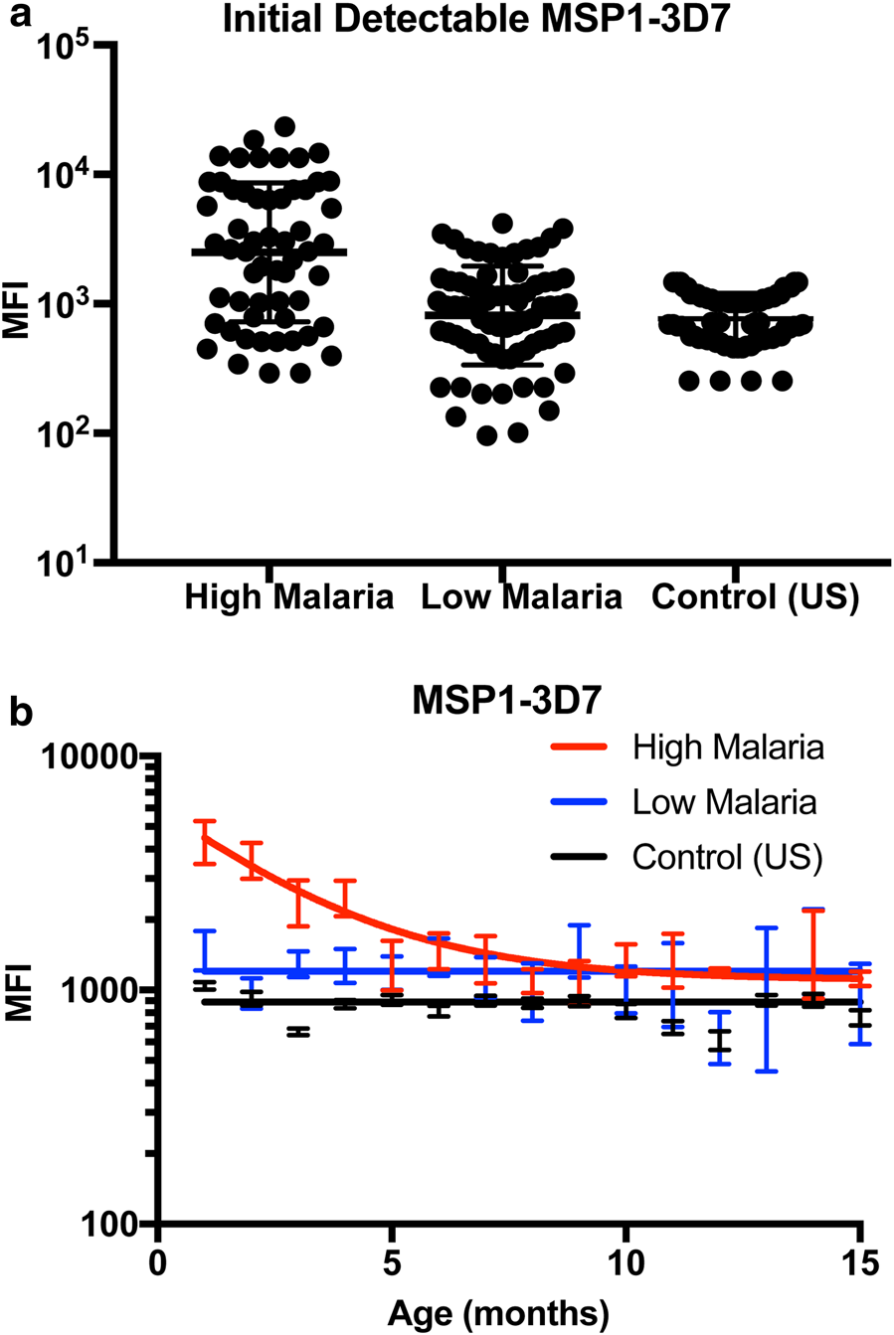
Kinetics of MSP1-3D7 IgG responses over time. **a** MSP1 3D7-specific antibodies were higher at first detectable value (around 1–2 months) in children living in a region of high malaria transmission. **b** Different trends in MSP1-3D7 IgG levels between children in high vs low malaria transmission region. MSP1-3D7 IgG drops in the first few months with an estimated a half-life of 1.75 months in children from the high malaria region. In the low malaria region (and uninfected adult US control), there was no clear trend of decreasing levels of maternal antibody. Error bars represent SEM

The children from the malaria^hi^ region had higher levels of MSP1-specific immunoglobulin at enrollment (P = 0.003; Fig. 2a). This is consistent with the assumed higher exposure of mothers from this region, and the correlation between maternal and neonatal antibodies [12, 21]. Importantly, although time-to-first infection did not differ between the high and low-malaria regions (P = 0.35; Fig. 1), the initial level of MSP1-specific antibody was a significant predictor of time-to-first detection of *P. falciparum* DNA, with those children with higher initial antibody levels showing longer times to detection of *P. falciparum* infection (HR = 0.77, per each log of MFI increase in MSP1-3D7 antibody binding; 95% CI = [0.63–0.95], P = 0.014 for MSP1-3D7, irrespective of region).

The higher initial antibody level and increased protection from antibodies observed in the malaria^hi^ children is a possible explanation as to why this group had lower risk of infection during the first 6 months, but it does not explain the greater risk of infection after 6 months (crossing of survival curves in Fig. 1). To better understand this observation, the dynamics of antibody levels with time were analysed further over the course of the study (Fig. 2b). As this study investigated time-to-first infection, the observed antibody dynamics can be considered as the dynamics of maternal antibodies in infants after birth. Although children in the malaria^hi^ group had higher initial antibody levels (at first detection), these levels decayed at a half-life of 1.75 months (95% CI = 0.94–5.92 months), until it returned to a plateau levels (1106 MFI, 95% CI = 193–2019 MFI) by approximately 8 months after birth. Therefore, it was likely that although children in the high malaria region experience an initial protective effect from maternal antibodies inherited from their frequently exposed mothers, this protection waned with time after which these children then experienced high exposure to *P falciparum* infection. In contrast, children in the low malaria region did not demonstrate significantly higher antibody levels than unexposed controls (P = 0.98, Additional file 5), and antibody levels did not decay with time (as they were already at baseline levels—1203 MFI; 95% CI = 1071–1334 MFI), suggesting that children in the low malaria region did not inherit temporary anti-malarial immunity from their mothers. As children from the high malaria region are likely to be more frequently exposed, this may explain why, after maternal antibodies reduce to baseline levels, the survival curves cross and these children were now at greater risk of infection compared to the low malaria group.

### The interplay between antibody protection and malaria exposure

To further understand the relationship between antibody mediated protection, risk of infection due to exposure, and time-to-infection, a number of additional Cox proportional hazards based survival models were investigated, including: a model with time dependent antibody level protection and models with threshold levels of antibody protection. These models showed no evidence for a specific threshold of antibody level where above this threshold the relationship between antibody level and protection was different to below the threshold (HR = 1.37 for the fold increase in HR before and after the threshold; P = 0.17). However, a model with continuous linear protection by antibody level describes this data better (a time-dependent Cox model, HR = 0.71 for each log of MFI increase in MSP1-3D7 antibody binding, p-0.054). The increased complexity of these Cox-based models failed to provide further insight possibly because MSP1 3D7 was not solely responsible for all causative protection, but rather serving as an indicator of overall protection inherited from the mother. Considering this, a parametric survival function (Eq. 1) was used to quantitatively describe the dynamics of time-to-first infection between these cohorts based on inherited protection rather than specific antibody level.

**Fig. 3:**
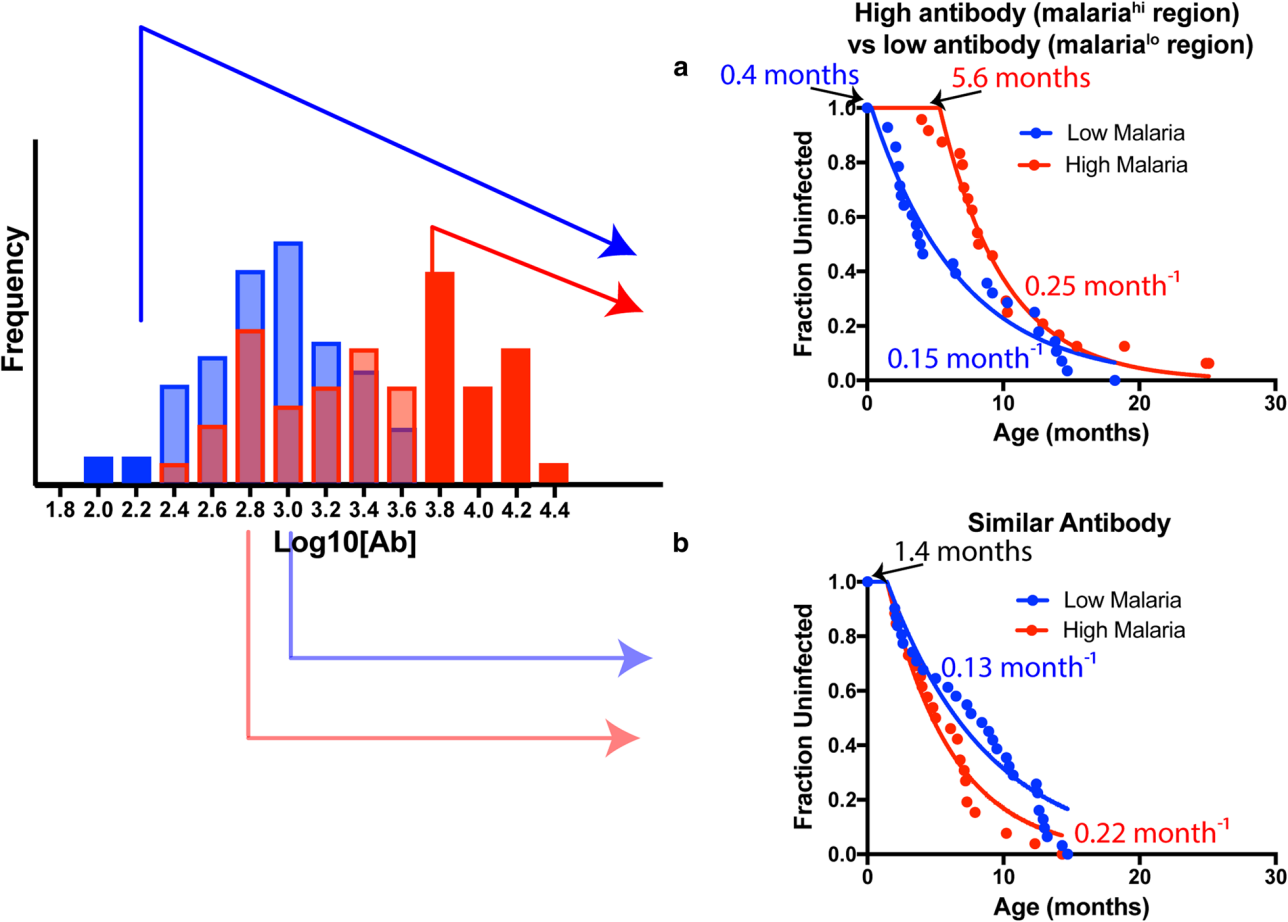
Time-to-first *Plasmodium falciparum* infection in Nandi vs Kisumu based on the observed levels of maternal antibody. Infants were divided into four groups. First, the two extremes of antibody levels were compared (**a**); Infants from a region of high malaria transmission with high levels of MSP1-3D7 antibody and infants from a low transmission region with low levels of MSP1-3D7 antibody. Modelling was used to estimate the duration of protective effect, and subsequent frequency of infection. There was evidence for a longer period of inherited immunity in the high group (presumably due to maternal antibody), but also a higher infection rate once immunity has decayed. The remaining children with more homogenous antibody levels, but from regions of different malaria exposure were also compared using a parametric model of duration of protective effect and subsequent infection rate in these two groups (**b**). There was no significant different in the predicted duration of protective effect between the two groups. Using a parametric survival analysis, assuming a constant rate of infection, the infection rate is higher in children from a region of high transmission

A custom survival model (Eq. 1) was fitted to this data that allows for different periods of inherited protection, and different frequencies of exposure. This model was used to compare the different risks of infection after adjusting for periods of inherited protection by including a delay term for when infection risk begins. To investigate the role of antibodies on this model, participants were divided into two separate subsets—(1) those with different initial antibody levels from each region (high antibody levels from malaria^hi^ and those with low antibody levels from malaria^lo^) and (2) those with similar initial antibody levels from each region (low antibody levels from malaria^hi^ and those with high antibody levels from malaria^lo^) (Fig. 3). Subset 1 was picked by first ranking children according to antibody levels at enrolment. This subset includes children with high levels (the highest 50%) from the malaria^hi^ group and children with low levels (the lowest 50%) from the malaria^lo^ group. Subset 2 was picked by including the lowest 50% from the malaria^hi^ group and the highest 50% from the malaria^lo^ group. Comparing children with divergent levels of initial inherited antibodies (subset 1), the delay to first infection was significantly higher in high malaria region compared to the low malaria region (5.6 months vs. 0.4 months in low malaria region, P < 0.0001, F-test). However, once the ‘protection’ waned (after the delay), the force of infection was higher for children from the high malaria region with high antibody levels (0.25 month^−1^ vs 0.15 month^−1^, P = 0.009, F-test—Fig. 3a).

Furthermore, children with an overlapping spectrum of MSP1 3D7 antibody levels from both high and low malaria regions (Fig. 3b) showed the opposite patterns of time to infection from that described above. That is, there was no significant difference in initial delay to infection between regions (1.21 months in malaria^lo^ area vs 1.64 months in malaria^hi^ area, shared value of 1.44 months, P = 0.21, F-test) after which there was a shorter time-to-first infection in the high malaria region (0.22 month^−1^ in malaria^hi^ vs 0.13 month^−1^ in malaria^lo^, P < 0.0001, F-test—Fig. 3b).

Taken together, these analyses show that the time to first infection curve in Fig. 1 can be decomposed into an initial period of inherited protection based on the level of initial (maternal) antibody from the high malaria region, followed by a subsequent higher infection rate.

## Discussion

This study shows that protective immunity through antibodies and the level of exposure to malaria infection can act as confounders in studies investigating malaria transmission rates. Although many studies have looked at the role of MSP1 IgG in *P. falciparum* infection [10, 14, 22, 23], only a few studies have looked at MSP1 IgG in infants with different levels of malaria exposure intensity in African settings. These have shown that repeated malaria infections and prolonged exposure is needed to maintain high levels of antibody in infants [24]. Additionally, the prevalence of MSP1-19 antibody is higher in high malaria transmission regions compared with low malaria transmission regions in the first few months of life, but these antibodies decline steadily over the first few months of life [25, 26]—similar to the observed dynamics of MSP1-3D7 in this study. However, one study also found evidence that even in a region with high malaria infection, the levels of antibody can remain low [27]. Moreover, inherited immunity in the form of cellular responses (as measured by interferon gamma responses to MSP1) in infants living in a low transmission decline rapidly without the presence of malaria infection [28].

Although there was strong evidence for higher levels of malaria exposure in Kisumu, unexpectedly the time-to-first-detection of *P. falciparum* DNA did not differ from that in Nandi, where there is relatively low exposure. It is currently believed that the level of maternal antibody is only useful as a marker of exposure, rather than protection [26]. However, these findings are uncertain as adjusting for the interaction between exposure, maternal antibody levels and infection can be extremely difficult [29]. This is a key advantage of this cohort and modelling approach where the confounding effects of inherited immunity (antibody level to MSP1) exposure to malaria, and time-to-first infection can be taken into account. Infants in high malaria regions frequently have higher initial antibody levels that confer protection until those antibody levels wane (approximately 6 months after birth). Following this those infants have an increased risk of subsequent infection due to their higher exposure. Similarly, infants whose parents were less exposed have no initial protection from infection, but also have a lower risk of infection due to less exposure.

One limitation of this study was that MSP1-3D7 and MSP1-FVO are unlikely to be solely responsible for the inherited protection of infants, and other antibodies and passive immunity are likely to play a role [14, 30]. MSP-1 antibody responses were chosen in this study because of their high levels of reactivity in children from the high malaria region and their association with time-to-infection. Thus, the selection filter may potentially have biased the observed associations between MSP-1 antibody level and risk of infection. However, the association of MSP-1 antibodies with protection has been previously observed, and the major insight of this work is to show how level of maternal malaria exposure may affect both antibody level and risk of infection, and how this plays out in early life. This limitation prevented more complex statistical models of the nature of inherited immunity to be explored. Exposure in these cohorts was assumed to be higher in Kisumu than in Nandi based on previous studies [15, 16], and the estimated infection rates after loss of maternal antibody protection suggest that the infection rate is around twofold higher in Kisumu (Fig. 3).

This study also relied on time to PCR-detectable infection. This is a stringent criterion, as parasites are frequently detectable by PCR even in heavily exposed and immune adults. Thus, it is likely that the level of maternal-derived antibody remaining at the time of PCR detection may still be sufficient to maintain parasitaemia to low levels. If protection from clinical illness has a much lower threshold of antibody level required, this may persist for a period after PCR-detectable infection is observed.

An additional limitation of this study is that other factors may also play a role in protection of infants from malaria, such as the nutritional effects of breast milk, high level of fetal haemoglobin (HbF), and different mosquito biting preference in infants vs adults (reviewed in [14]). Because these differences were not captured in this cohort, it remains unknown how they might affect the risk for acquisition of malaria in the infants.

The strengths of this study are the nature of the cohorts and rigorous quantitative analyses that can be used to untangle the effects of inherited (but decaying) antibody immunity and exposure. These findings set the framework for future cohort studies of malaria immunity by demonstrating how the exposure-outcome relationship is confounded by the presence of malaria immunity and malaria exposure and, therefore, exposure needs to be taken into account for accurate estimation of antibody driven protection. However, accurate measurement of individual risk of malaria exposure is not trivial. Many studies attempt to adjust for the heterogeneity in exposure, but this was mainly estimated with a proxy variable such as distance to water, baseline of antibody concentration, or ethnicity (reviewed in [29]). Without this adjustment, conflicting results on the nature of antibody driven malaria protection will continue in cohort studies.

Overall, this study demonstrates the need to disentangle the complex relationship between transmission intensity, antibody responses, and observed time-to-infection to correctly identify protective immune responses in *P. falciparum* infection.

## Additional files

**Additional file 1.** Distribution of the first time of sampling.

**Additional file 2.** Cox proportional hazard regression using various antibodies to predict time to first malaria infection (detection by PCR).

**Additional file 3.** Risk table for infants living in Kisumu (malaria^hi^ region).

**Additional file 4.** Risk table for infants living in Nandi (malaria^lo^ region).

**Additional file 5.** Comparing the levels of first detectable antibody in high malaria, low malaria, and uninfected US adult control.

**Additional file 6.** Kinetics of MSP1-FVO IgG.

**Additional file 7.** Time-to-first *P. falciparum* infection in Nandi vs Kisumu based on the observed levels of maternal antibody (MSP1-FVO).
