# Supplementary figures and images for: Interaction between maternally derived antibodies and heterogeneity in exposure combined to determine time-to-first Plasmodium falciparum infection in Kenyan infants
Source: Malar J. 2019 Jan 22;18:19. doi: 10.1186/s12936-019-2657-6 (PMC6343364; doi:10.1186/s12936-019-2657-6)

Additional file 1

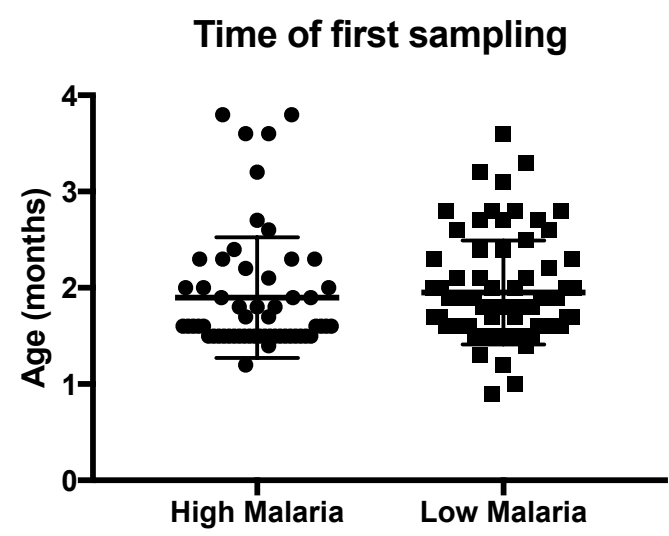

Additional file 1. Distribution of the first time of sampling

Supplement: Supplementary file 1 — Additional file 1. Distribution of the first time of sampling. [file 12936_2019_2657_MOESM1_ESM.pdf]
