# Supplementary material for: Interaction between maternally derived antibodies and heterogeneity in exposure combined to determine time-to-first Plasmodium falciparum infection in Kenyan infants
Source: Malar J. 2019 Jan 22;18:19. doi: 10.1186/s12936-019-2657-6 (PMC6343364; doi:10.1186/s12936-019-2657-6)
Supplement: Supplementary file 2 — Additional file 2. Cox proportional hazard regression using various antibodies to predict time to first malaria infection (detection by PCR). [file 12936_2019_2657_MOESM2_ESM.pdf]

Additional file 2. Comparing the levels of first detectable antibody in high malaria, low malaria, and uninfected US adult control

Name	High Malaria vs Control			Low Malaria vs Control			High Malaria vs Low Malaria		
	Actual Diff.	95%CI	p-val*	Actual Diff.	95%CI	p-val*	Actual Diff.	95%CI	P-val*
MSPDBL1	1933	[1615; 2184]	<0.0001	1758	[1170; 1758]	<0.0001	320	[27; 938]	0.04
MSPDBL2	1103	[568; 1510]	<0.0001	242	[73; 442]	0.006	1218	[736; 1624]	<0.0001
AMA-3D7	2676	[2173; 3440]	<0.0001	247	[-135; 293]	0.4	3016	[1728; 3404]	<0.0001
AMA-FVO	3581	[2497; 4386]	<0.0001	27	[-204; 235]	0.81	3661	[2692; 4584]	<0.0001
CEITOS	2287	[1546; 2240]	<0.0001	1811	[1182; 2349]	<0.0001	628	[168; 960]	0.005
CSP	2489	[1771; 2496]	<0.0001	2944	[2076; 3089]	<0.0001	-547	[-896; -162]	0.002
EBA-140	439	[273; 546]	<0.0001	601	[453; 843]	<0.0001	-89	[-354; -6]	0.03
EBA-175 W2mef	994	[753; 1191]	<0.0001	2117	[1646; 2294]	<0.0001	231	[77; 451]	0.007
EBA-175 3D7	930	[643; 998]	<0.0001	1180	[830; 1187]	<0.0001	645	[438; 953]	<0.0001
EBA-181	107	[-14; 347]	0.07	482	[247; 550]	<0.0001	291	[75; 371]	0.008
LSA1	377	[129; 556]	0.004	283	[42; 417]	0.02	-26	[-283; 124]	0.43
MSP1-3D7	1914	[1042; 2631]	<0.001	132	[-220; 159]	0.98	1682	[696; 2695]	<0.0001
MSP1-FUP	232	[97; 368]	0.003	359	[-30; 497]	0.10	-214	[-322; 103]	0.31
MSP1-FVO	986	[551; 1773]	<0.0001	75	[-157; 175]	0.74	1061	[428; 1642]	<0.0001
MSP2-FC27	3494	[2870; 3479]	<0.0001	4891	[4256; 5948]	<0.0001	194	[-89; 371]	0.27
MSP6	984	[749; 1000]	<0.0001	846	[492; 823]	<0.0001	22	[-89; 119]	0.79
MSP7	981	[845; 1203]	<0.0001	1105	[694; 1123]	<0.0001	69	[-82; 195]	0.36
MSP3	262	[0.91; 485]	0.054	242	[73; 442]	0.006	9	[-220; 249]	0.93
Rh4.9	-2985	[-4413; -1286]	<0.0001	1599	[745; 1696]	<0.0001	-2735	[-4898; -1296]	<0.0001
Rh2	369	[77; 610]	0.015	724	[523; 941]	<0.0001	-740	[-863; -289]	<0.0001
RIPR CT	2623	[2016; 2695]	<0.0001	3251	[2610; 3817]	<0.0001	88	[-173; 354]	0.53
RIPR NT	3083	[2325; 3238]	<0.0001	4317	[3369; 4856]	<0.0001	-502	[-905; -106]	0.01
SERA5	208	[22; 230]	0.04	1559	[469; 1841]	<0.0001	276	[-26; 291]	0.11

*p-val was determined based on the significance of the Mann-Whitney test (not corrected for multiple comparison).
