# Supplementary material for: Interaction between maternally derived antibodies and heterogeneity in exposure combined to determine time-to-first Plasmodium falciparum infection in Kenyan infants
Source: Malar J. 2019 Jan 22;18:19. doi: 10.1186/s12936-019-2657-6 (PMC6343364; doi:10.1186/s12936-019-2657-6)
Supplement: Supplementary file 3 — Additional file 3. Risk table for infants living in Kisumu (malariahi region). [file 12936_2019_2657_MOESM3_ESM.pdf]

Additional file 3. Risk table for infants living in HM region

Age (months)	People at risk<br>High Malaria
0	57
1.5	57
2	56
2.1	52
3	51
3.6	47
3.9	46
4	42
4.4	40
4.5	39
4.7	38
4.8	37
5	36
5.5	34
6.1	33
6.7	32
6.8	31
7	29
7.1	28
7.2	25
7.3	22
7.4	20
7.9	19
8.1	18
8.2	16
9.2	15
10.2	14
10.3	8
11.1	7
11.4	6
12.3	5
14.1	4
14.2	3
15.4	1
