# Supplementary material for: Interaction between maternally derived antibodies and heterogeneity in exposure combined to determine time-to-first Plasmodium falciparum infection in Kenyan infants
Source: Malar J. 2019 Jan 22;18:19. doi: 10.1186/s12936-019-2657-6 (PMC6343364; doi:10.1186/s12936-019-2657-6)
Supplement: Supplementary file 4 — Additional file 4. Risk table for infants living in Nandi (malarialo region). [file 12936_2019_2657_MOESM4_ESM.pdf]

Additional file 4. Risk table for infants living in LM region

Age (months)	People at risk<br>Low Malaria
0	74
1.5	74
2	74
2.1	68
2.2	64
2.3	63
2.4	59
2.5	57
2.6	55
2.7	53
3.3	52
3.6	50
3.7	48
3.9	47
4.1	46
4.9	45
5	43
5.9	42
6.3	41
6.5	39
7.3	38
7.6	37
7.7	36
7.9	35
8.3	33
8.4	32
8.8	31
8.9	30
9.2	29
9.4	28
9.5	27
9.6	26
10.1	25
10.2	24
10.3	22
10.4	20
10.6	19
10.7	18
11.3	17
12.3	16
12.4	15

12.5	14
12.6	13
12.9	12
13.2	11
13.8	10
13.9	9
14.3	8
14.7	6
14.8	5
15.2	3
15.3	2
18.2	1
