# Supplementary material for: Interaction between maternally derived antibodies and heterogeneity in exposure combined to determine time-to-first Plasmodium falciparum infection in Kenyan infants
Source: Malar J. 2019 Jan 22;18:19. doi: 10.1186/s12936-019-2657-6 (PMC6343364; doi:10.1186/s12936-019-2657-6)
Supplement: Supplementary file 5 — Additional file 5. Comparing the levels of first detectable antibody in high malaria, low malaria, and uninfected US adult control. [file 12936_2019_2657_MOESM5_ESM.pdf]

Additional file 5. Cox proportional hazard regression using various antibodies to predict time to first malaria infection (detection by PCR)

Name	High Malaria			Low Malaria			Combined		
	HR	95%CI	p-val*	HR	95%CI	p-val*	HR	95%CI	P-val*
MSPDBL1	0.96	0.53 - 1.74	0.91	1.29	0.87 - 1.91	0.21	1.09	0.81 - 1.48	0.56
MSPDBL2	0.87	0.53 - 1.41	0.57	1.05	0.72 - 1.54	0.79	0.95	0.74 - 1.22	0.70
AMA-3D7	1.26	0.89 - 1.77	0.19	0.97	0.73 - 1.28	0.83	1.03	0.86 - 1.22	0.77
AMA-FVO	1.26	0.89 - 1.79	0.19	0.96	0.73 - 1.27	0.78	1.01	0.85 - 1.19	0.94
CEITOS	1.05	0.76 - 1.45	0.77	0.89	0.62 - 1.27	0.52	1.02	0.81 - 1.28	0.84
CSP	0.87	0.63 - 1.19	0.38	1.06	0.77 - 1.45	0.73	0.97	0.77 - 1.16	0.62
EBA-140	0.61	0.38 - 0.97	0.04	1.16	0.87 - 1.55	0.32	0.95	0.76 - 1.20	0.68
EBA-175 W2mef	0.87	0.62 - 1.23	0.44	1.15	0.79 - 1.65	0.47	1.00	0.80 - 1.26	0.99
EBA-175 3D7	0.76	0.51 - 1.13	0.18	1.06	0.80 - 1.38	0.70	0.93	0.77 - 1.13	0.46
EBA-181	0.75	0.52 - 1.04	0.08	1.00	0.73 - 1.38	1.00	0.92	0.73 - 1.15	0.45
LSA1	0.95	0.64 - 1.42	0.82	1.14	0.80 - 1.62	0.46	1.04	0.81 - 1.34	0.75
MSP1-3D7	0.58	0.41 - 0.82	<0.0001	0.83	0.58 - 1.08	0.12	0.77	0.63 - 0.95	0.01
MSP1-FUP	0.57	0.39 - 0.83	<0.0001	0.76	0.55 - 1.05	0.10	0.67	0.53 - 0.85	<0.0001
MSP1-FVO	0.69	0.51 - 0.92	0.01	0.82	0.58 - 1.06	0.10	0.74	0.61 - 0.91	<0.0001
MSP2-FC27	0.87	0.55 - 1.38	0.56	0.95	0.69 - 1.31	0.76	0.94	0.73 - 1.22	0.65
MSP6	0.77	0.47 - 1.25	0.29	1.06	0.67 - 1.68	0.81	0.93	0.66 - 1.30	0.66
MSP7	0.94	0.67 - 1.30	0.69	1.08	0.75 - 1.55	0.69	1.01	0.80 - 1.28	0.93
MSP3	0.68	0.46 - 1.01	0.06	1.03	0.70 - 1.51	0.88	0.81	0.62 - 1.07	0.14
Rh4.9	0.83	0.64 - 1.08	0.18	0.88	0.65 - 1.18	0.39	0.86	0.73 - 1.02	0.08
Rh2	0.82	0.56 - 1.19	0.29	0.91	0.63 - 1.31	0.61	0.90	0.71 - 1.13	0.36
RIPR CT	0.85	0.57 - 1.24	0.39	1.20	0.83 - 1.74	0.33	1.02	0.79 - 1.32	0.87
RIPR NT	1.01	0.70 - 1.44	0.97	0.97	0.640- 1.47	0.89	1.02	0.79 - 1.3	0.87
SERA5	0.92	0.59 - 1.43	0.71	0.99	0.67 - 1.46	0.95	0.98	0.74 - 1.32	0.92

*p-val was determined based on the significance of the hazard ratio (proportional Cox regression – not corrected for multiple comparison).
