# Supplementary material for: Interaction between maternally derived antibodies and heterogeneity in exposure combined to determine time-to-first Plasmodium falciparum infection in Kenyan infants
Source: Malar J. 2019 Jan 22;18:19. doi: 10.1186/s12936-019-2657-6 (PMC6343364; doi:10.1186/s12936-019-2657-6)
Supplement: Supplementary file 6 — Additional file 6. Kinetics of MSP1-FVO IgG. [file 12936_2019_2657_MOESM6_ESM.pdf]

Additional file 6

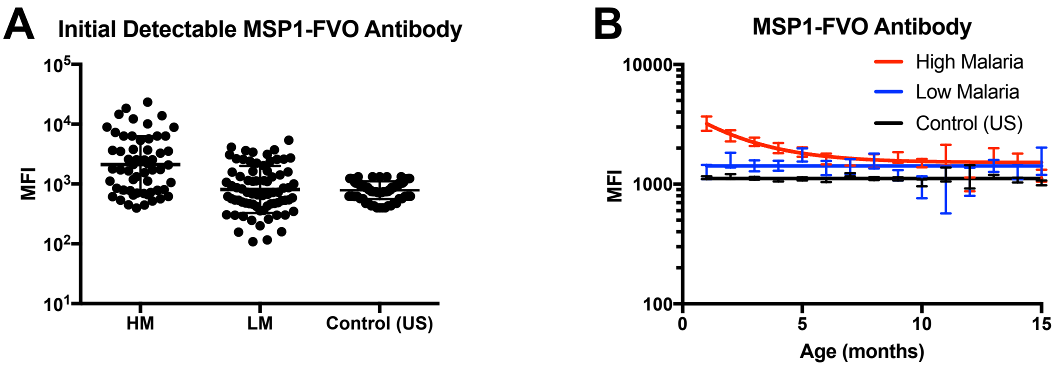

Additional file 6. Kinetics of MSP1-FVO IgG. (A) MSP1 FVO- specific antibodies were higher at first detectable value (around 1-2 months) in children living in a region of high malaria transmission. (B) Different trends in MSP1-FVO IgG levels between children in high vs low malaria transmission region. MSP1-FVO IgG drops in the first few months with an estimated a half-life of 1.5 months in children from the high malaria region. In the low malaria region (and uninfected adult US control), there was no clear trend of decreasing levels of maternal antibody. Error bars represent SEM.
