# Supplementary material for: Interaction between maternally derived antibodies and heterogeneity in exposure combined to determine time-to-first Plasmodium falciparum infection in Kenyan infants
Source: Malar J. 2019 Jan 22;18:19. doi: 10.1186/s12936-019-2657-6 (PMC6343364; doi:10.1186/s12936-019-2657-6)
Supplement: Supplementary file 7 — Additional file 7. Time-to-first P. falciparum infection in Nandi vs Kisumu based on the observed levels of maternal antibody (MSP1-FVO). [file 12936_2019_2657_MOESM7_ESM.pdf]

Additional file 7

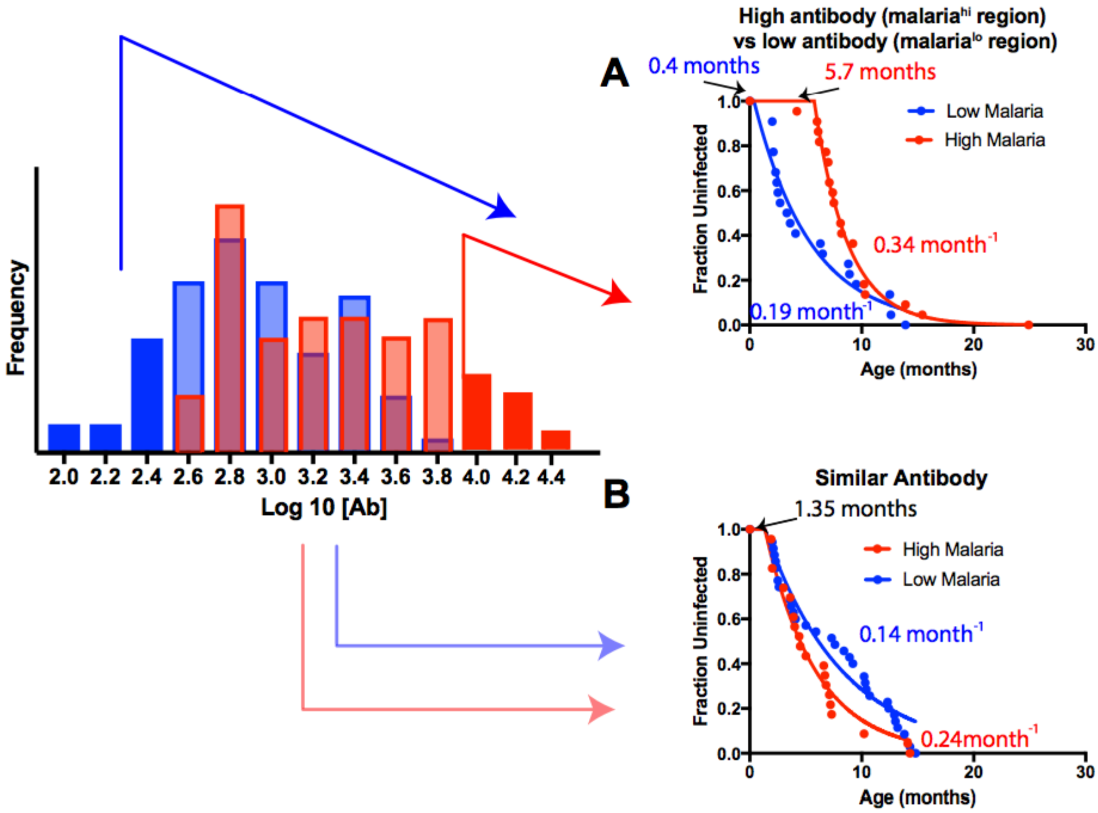

Additional file 7. Time-to-first *P. falciparum* infection in Nandi vs Kisumu based on the observed levels of maternal antibody (MSP1-FVO). We subdivided infants into four groups. We first compared the two extremes of antibody levels (panel A), infants from a region of high malaria transmission with high levels of MSP1-FVO and infants from a low transmission region with low levels of MSP1-FVO, and compared the time-to-first infection. We found evidence for a longer period of inherited immunity in the high group (due to presumably maternal antibody), and also a higher infection rate once immunity has decayed. We then compared two groups with homogenous antibody levels, but from regions of different malaria exposure (panel B). The infants from a region of high malaria transmission and children from a low transmission region were compared to see the time-to-first infection and subsequent infection rate between these two groups. We found no significant different in time-to-first infection between the two groups. Using a parametric survival analysis, assuming a constant rate of infection, we found that the infection rate is higher in children from a region of high transmission.
